# Cytochrome P450 1B1 polymorphism drives cancer cell stemness and patient outcome in head-and-neck carcinoma

**DOI:** 10.1038/s41416-020-0932-5

**Published:** 2020-06-22

**Authors:** Valérie Le Morvan, Élodie Richard, Maud Cadars, Delphine Fessart, Léa Broca-Brisson, Céline Auzanneau, Alban Pasquies, Anouchka Modesto, Amélie Lusque, Simone Mathoulin-Pélissier, Amélie Lansiaux, Jacques Robert

**Affiliations:** 1 INSERM Unit 1218, Université de Bordeaux, Bordeaux, France; 2 Institut Universitaire du Cancer de Toulouse, Toulouse, France; 3 INSERM Unit 1219, Université de Bordeaux, Bordeaux, France

**Keywords:** Cancer, Genetics

## Abstract

**Background:**

Cytochrome P450 1B1 (CYP1B1) is mostly expressed in tumours and displays unusual properties. Its two polymorphic forms were differently associated with anticancer drug sensitivity. We decipher here the role of this polymorphism in anticancer drug efficacy in vitro, in vivo and in the clinical setting.

**Methods:**

From head-and-neck squamous cell carcinoma cell lines not expressing CYP1B1, we generated isogenic derivatives expressing the two forms. Proliferation, invasiveness, stem cell characteristics, sensitivity to anticancer agents and transcriptome were analysed. Tumour growth and chemosensitivity were studied in vivo. A prospective clinical trial on 121 patients with advanced head-and-neck cancers was conducted, and a validation-retrospective study was conducted.

**Results:**

Cell lines expressing the variant form displayed high rates of in vitro proliferation and invasiveness, stemness features and resistance to DNA-damaging agents. In vivo, tumours expressing the variant CYP1B1 had higher growth rates and were markedly drug-resistant. In the clinical study, overall survival was significantly associated with the genotypes, wild-type patients presenting a longer median survival (13.5 months) than the variant patients (6.3 months) (*p* = 0.0166).

**Conclusions:**

This frequent CYP1B1 polymorphism is crucial for cancer cell proliferation, migration, resistance to chemotherapy and stemness properties, and strongly influences head-and-neck cancer patients’ survival.

## Background

The screening of gene polymorphisms associated with chemosensitivity in two panels of cancer cell lines, originating from the National Cancer Institute (NCI-60) and the Japanese Foundation for Cancer Research (JFCR-45), allowed the identification of a close association between a single-nucleotide polymorphism (SNP) of cytochrome P450 1B1 (CYP1B1) (V432L, rs1056836) and in vitro sensitivity to several anticancer agents.^1^ This polymorphism had been shown to be significantly associated with the risk of several cancer types,^2–4^ with poor prognosis of prostate cancer patients treated with docetaxel^5^ and to lower the response rate of patients treated for breast cancer in the neoadjuvant setting with the FEC combination (fluorouracil, epirubicin and cyclophosphamide).^6^ These features can hardly be related to the catalytic activity of CYP1B1 since this CYP does not metabolise any of the drugs used in these clinical studies.

Cytochrome P450 1B1 (CYP1B1) is an unusual cytochrome whose functions are still controversial. Unlike most CYPs, it displays low expression levels in normal tissues, but is frequently overexpressed in tumours.^7,8^ It has been even considered as a “universal tumor antigen”^9^ and was included in a list of cancer antigen prioritisation for targeted therapies by the National Cancer Institute (NCI).^10^ As all CYPs, CYP1B1 is able to catalyse oxidation reactions, and its main known substrates are oestradiol and polyaromatic carcinogens such as benzopyrene.^8^ However, these catalytic functions cannot explain its characteristic overexpression in tumour tissues. In addition, its expression has been associated with resistance to docetaxel^11^ and daunorubicin^12^ in in vitro models, and these features cannot either be explained by its enzymatic activity towards anticancer drugs.

In order to understand the role of CYP1B1 and of its common polymorphism V432L, we characterised head-and-neck squamous cell carcinoma (HNSCC) cell lines that do not express this cytochrome, and we generated isogenic derivatives of these lines expressing similar levels of the two variant forms of CYP1B1. Our study reveals that the variant (L^432^) CYP1B1 form is a strong enhancer of cell proliferation, both in vitro and in vivo, and of migration and invasion capacity in vitro. It is also associated with resistance to DNA-damaging agents in vitro and in vivo. Transcriptome analysis by RNA-seq as well as protein blots showed that the cells expressing the variant CYP1B1 genotype presented an accentuated epithelial character as compared with those expressing the wild-type form, in agreement with in silico data extracted from cell line collections. The variant CYP1B1 cell cultures are enriched in ALDH-positive cells as compared with wild-type CYP1B1 cell cultures, which could be related to the phenotypic differences detected. Finally, in order to identify associations between germline CYP1B1 polymorphism and patients’ survival, a clinical study was done, including in advanced HNSCC patients treated with chemotherapy and cetuximab. This prospective study revealed that the variant CYP1B1 genotype was associated with poor prognosis, and was confirmed in a retrospective validation study.

## Methods

### Cell culture

CAL27 and CAL33 HNSCC cell lines^13^ were kindly provided by Dr J.L. Fischel (Centre Antoine Lacassagne, Nice, France, where they were initially obtained). They were authenticated by Azur Génétique (Nice, France) as identical to the corresponding DSMZ cell lines (report reference AGLC-14-00225). They were cultured in Dulbecco’s modified Eagles’s medium (Invitrogen, Cergy-Pontoise, France) supplemented with 10% heat-inactivated foetal calf serum (Pan Biotech) and maintained at 37 °C in a humidified atmosphere containing 5% CO_2_ in Petri dishes or flasks of various sizes. They were replicated every 4–5 days, and the medium was changed once in-between.

### Genotyping

DNA was extracted using the QiaAmp mini kit for cells and the QiaAmp Blood kit for blood samples. The CYP1B1 V432L variation (rs1056836) was determined by pyrosequencing. Direct sequencing of PCR fragments without any further purification was carried out on the Pyrosequencer PyroMark ID system (Qiagen) according to the instructions of the manufacturer. The primers used were as follows: sense, 5′ dR-Biotin-CTACCACATTCCCAAGGACACT 3′; antisense, 5′ GCTGGTCAGGTCCTTGTTG 3’; pyrosequencing, 5’ CGGGTTAGGCCACTT 3’.

### CYP1B1 promoter methylation analysis

Quantitative methylation was evaluated by pyrosequencing on two CpG islands, located at two separate locations in 5’ and 3’ of the promoter, and containing 6 and 5 CpG dinucleotide sequences, respectively. Genomic DNA was extracted using the DNeasy Blood and Tissue Kit (Qiagen), according to the instructions of the manufacturer. DNA samples (500 ng), including positive controls for methylated and non-methylated status, were treated with sodium bisulfite using the Epitect Bisulfite Kit (Qiagen) as described by the manufacturer. Bisulfite-modified DNA was then used as a template for PCR amplification. Amplification and sequencing primers were designed for the bisulfite-converted DNA using PyroMark Assay Design Software 2.0 (Qiagen). For each amplification reaction, one primer was labelled with 5′-biotin synthesised with the standard phosphoramidite method and purified by reversed-phase HPLC.

The PCRs were performed in a volume of 50 μL with 1 × PCR buffer, 0.2 mM of dNTPs, 1.5 mM of MgCl_2_, 2 μl of bisulfite-converted DNA (corresponding to 1 μg of the initial DNA amount), 5 pmoles of each primer and 0.2 units of Taq DNA Polymerase (Qiagen). Initial denaturation was carried out for 3 min at 94 °C, followed by 50 PCR cycles (94 °C for 20 s, appropriate Tm for 20 s, 72 °C for 30–45 s) and final extension for 7 min at 72 °C. The sets of primers used for PCR and sequencing were designed by Qiagen. The first set amplified a fragment of 96 pb containing 6 CpG in the 5′ promoter region, and the second set amplified a fragment of 166 pb containing 5 CpG in the 3′ promoter region. Pyrosequencing was performed using the PyroGold Kit and a PSQ 96 ID instrument (Qiagen) as described by the manufacturer. CpG site methylation quantification was done using the methylation software Pyro Q-CpG 2.0 (Qiagen). The coefficient of variance for the methylation level of CpG sites was generally <2%.

### Cloning and infection

#### Cloning vector

The wild-type human CYP1B1 cDNA was purchased from Open Biosystems, and was cloned in a pDONR201vector using the Gateway Cloning kit (Invitrogen). An attB-flanked PCR product of CYP1B1 cDNA was cloned into the pDONR201 entry vector and then transferred into destination vector pSD-69 using the Gateway Cloning kit (Invitrogen). The pSD-69 plasmid contains the human phosphoglycerate kinase (PGK) promoter, a Gateway attR cassette (Invitrogen), the mouse PGK promoter and the puromycin acetyltransferase gene cloned into pRRLhPGK.GFP.SIN18.^14^ The variant cDNA was obtained by site-directed mutagenesis through replacement of G^1697^ by C^1697^, using the Site-Directed Mutagenesis kit (Stratagene). Constructions were verified by sequencing, and nucleotide replacement was checked by pyrosequencing. The pER51CYP1B1-WT (G allele: WT) and pER51CYP1B1-VAR (C allele: VAR) plasmid DNAs were then used for the generation of viruses and the infection of cell lines. The tdTomato-CAL27 and -CAL33 isogenic cell lines were produced by transduction of the cells with a second lentivirus carrying the tdTomato reporter gene (PGK-tdTomato), which was a generous gift from Pr François Moreau-Gaudry (University of Bordeaux).

#### Virus production

The CYP1B1 viruses were produced by co-transfection of human kidney 293T cells. 293T cells (4.4 × 10^6^) were plated, transfected on the following day with the packaging construct psPAX2 (10 μg) (Addgene), together with the vesicular stomatitis virus glycoprotein envelope vector pSD11 (4 μg) (a gift from Dr. Elodie Richard, INSERM) and a vector construct (10 μg of pER51CYP1B1-WT, pER51CYP1B1-VAR or pER15 control vector [pER51-GFP, CYP1B1 cDNA being replaced by GFP]) using calcium phosphate DNA precipitation and HeBS buffer (Hepes Buffered Saline) (Sigma-Aldrich). After 40 h, viral supernatants were collected in culture medium, filtered through 0.22-μm filters and immediately stored at −80 °C.

#### Infection

Two different HNSCC cell lines with no detectable CYP1B1 expression, CAL27 and CAL33, were infected. In total, 2 × 10^5^ cells were infected with viruses bearing the pER51CYP1B1-WT vector, or the pER51CYP1B1-VAR vector, or the pER15 control vector (p15), at a multiplicity of infection (MOI) of 10 or 40. Cells were then washed and amplified in 25-cm^2^ flasks, and selected by puromycin (2 µg/ml) for further analysis.

### In vitro assays

All in vitro assays were performed at least three times in triplicate.

#### Cell-proliferation assay

To assess proliferation, cells were plated on 60-mm Petri dishes at the density of 10^5^ cells per dish, and the medium was changed every 2 days. After trypsinisation, cell counts were performed with the aid of a haemocytometer using Trypan blue exclusion to monitor viability.

#### Cloning efficiency in agar

Cloning efficiency of the isogenic cell lines was evaluated in 24-well plates covered with 500 μl of 0.6% agar onto which 10^3^ cells suspended in 250 μl of 0.4% agar-containing culture medium were seeded. After incubation at 37 °C in a 5% CO_2_ atmosphere for 7 days, the colonies constituted by at least 50 cells were counted visually.

#### Wound-healing assay

For the scratch-wound assays, 4–6 × 10^5^ cells were plated on 60-mm Petri dishes and allowed to reach 70–80% of confluence (2 days). After medium elimination, cell layers were wounded using a 1-ml micropipette tip. Cells plated on the culture dishes were rinsed with PBS twice, and to differentiate the contributions of cell proliferation and migration to wound closure, the cell cycle blocker hydroxyurea (0.5 mM) was added in the medium. Wound closure was monitored by still photography at 0, 24 h and 48 h after wounding. Quantification of wound-healing assay was performed by measuring the percentage of wound area remaining at 24 or 48 h using ImageJ software. Three independent experiments were performed in duplicate.

#### Transmigration assays

The transmigration assays were performed using 24-well Transwell dishes (Falcon). Cells (2 × 10^4^) were resuspended in 200 μL of serum-free DMEM medium and placed in the top chambers; 800 μl of DMEM medium supplemented with 10% FBS was added to the bottom chambers. After 0, 4 h, 6 h and 12 h of incubation at 37 °C, non-invading cells were removed from the top chambers with a cotton swab. The invading cells at the bottom surface of the filter were fixed in 3% paraformaldehyde and stained with Giemsa (Sigma-Aldrich) for 30 min. The invading cells were visualised at ×100 magnification and counted with ImageJ software (Plugin Cell Counter) on the entire insert for each filter.

#### Drug-sensitivity assay

Drug cytotoxicity was evaluated by the MTT (3-(4,5-dimethylthiazole-2-yl)-2,5-diphenyl tetrazolium bromide) assay. Cells in exponential growth were trypsinised and resuspended in fresh culture medium to give a concentration of 10^3^ cells per 200 μl. The cell suspensions were seeded at 1 × 10^3^ cells per well in 96-well plates and left to attach in a 5% CO_2_ incubator at 37 °C. After incubation, the medium was replaced with fresh culture medium supplemented by drugs at appropriate concentrations. After 72 h of incubation at 37 °C, cell survival was estimated by MTT coloration at 570 nm. IC_50_ values were obtained from the cytotoxicity curves by interpolation. Fold resistance was calculated as the ratio between IC_50_ values in control and isogenic cells. Statistical analysis was performed using ANOVA. All assays were performed three times in triplicate.

#### RNA-seq transcriptome analysis

Cells were washed twice with phosphate-buffered saline (PBS). RNA was extracted using the RNeasy blood and Tissue kit (Qiagen) according to the manufacturer’s instructions. The quantity and quality of each RNA sample were measured spectrophotometrically using Nanodrop 1000 (Thermo Scientific, Waltham).

Transcriptome analysis by RNA sequencing was realised by Integragen [www.integragen.com]. Briefly, the libraries were prepared according to the protocol Truseq Stranded mRNA kit according to the instructions of the supplier. After capture of the polyA mRNAs from 5 mg of total RNAs, they were fragmented to about 400 b and retrotranscribed to cDNAs. After ligation and amplification by PCR, sequencing was performed as paired ends on 100 b on an Illumina HiSeq sequencer. The bioinformatics analysis performed by Integragen was performed according to Trapnell et al.^15^ in 4 steps: (1) sequence alignment on the reference genome (hg19) using the TopHat2 software, (2) detection assembly and quantification of the transcripts with the Cufflinks software, (3) transcript annotation by comparison with the RefSeq annotation using the CuffCompare software and (4) detection of fusion transcripts with the TopHat-Fusion software.

#### Protein analyses

Cells were washed twice in 1 ml of phosphate-buffered saline (PBS) at 4 °C, and were centrifuged for 5 min at 3000 × *g*. Cell pellets were lysed in Tris-HCl buffer (50 mM, pH 8) containing NaCl 150 mM, NP40 1%, sodium deoxycholate 1%, CaCl_2_ 1 mM and a protease inhibitor cocktail (Roche). After 30 min at 4 °C, the samples were centrifuged for 15 min at 30,000 × *g*. The supernatants containing soluble proteins were analysed by western blotting. Thirty micrograms of proteins were separated by 10% sodium dodecyl sulfate–polyacrylamide gel electrophoresis and transferred onto nitrocellulose membranes (GE Amersham), using liquid transfer. Membranes were then blocked for 1 h in 5% skim milk in Tris-buffered saline-T (Tris-HCl, pH 7.5, 100 mM, NaCl 0.9% and Tween-20 0.05%) and incubated overnight at 4 °C with primary antibodies against ESRP1, RAB25, GNB4 and CDH2 (N-cadherin) (all diluted at 1:500, Atlas antibodies, Ozyme), and peroxidase-conjugated secondary anti-rabbit or anti-mouse antibodies. GAPDH (Millipore antibody, diluted at 1:50,000) was used as a protein-loading reference control in each cell line. After washes, signals were detected using the Enhanced Chemiluminescence Reagent (Millipore).

### In vivo studies

#### Animals

The ARRIVE guidelines were followed all along the in vivo experimentation. NSG (NOD/SCID gamma) mice were produced and housed at the University of Bordeaux animal facility (Institutional agreement number A33063916), following the rules and regulations governed and enforced by the Institutional Animal Care and Use Committee. Eight- to 10-week-old animals were included in the protocols. Mice were monitored weekly for body weight, and were also examined for aspect and behaviour during the time course of the experiments. Anaesthesia was carried out with isoflurane (induction: 5% at a flow rate of 2 l/min, maintenance: 2% at a flow rate of 2 l/min), in sterile conditions. At the end of the experiments, mice were killed by cervical dislocation.

#### Tumour xenografts

In order to evaluate tumour growth in vivo, groups of 5 female mice were anaesthetised with isoflurane, and 1 × 10^6^ cells (CAL27 lines) or 5 × 10^6^ cells (CAL33 lines) suspended in 100 μl of culture medium were injected subcutaneously into their right flank, and two independent experiments were performed. For the evaluation of drug activity, groups of 4 female mice were injected subcutaneously with 1 × 10^6^ cells (CYP1B1-VAR line) or 3 × 10^6^ cells (CYP1B1-WT and p15 lines). At selected times after tumour grafting, mice were anaesthetised with isoflurane and placed in a special imaging chamber of a photon bioimager (Biospace Lab, Paris, France); tumour growth was evaluated by quantification of fluorescence, thanks to the expression of the tdTomato gene. Signals were quantified with the M3Vision software (Biospace Lab) within defined regions of interest of identical dimensions. A control tumour growth experiment was conducted in a group of male mice in order to detect a possible effect of gender.

### Stemness features

CAL27- and CAL33-derived CYP1B1-WT and CYP1B1-VAR cells were stained with ALDH reagents (Aldefluor, Stemcell Technologies Inc). ALDH^+^ and ALDH^−^ cells were sorted with a FACSAria Cell Sorting System (BD Biosciences) and resuspended in 100 μL of 3:1 PBS:Matrigel (BD Biosciences). In vivo studies were then performed; 3,000–300 for CAL27 CYP1B1-WT and 3000–10 for CAL27 CYP1B1-VAR FACS-sorted cells were injected subcutaneously into the flanks of 6- to 8-week-old female NSG mice; the right flank of the mouse received the ALDH^+^ cells, whereas the left flank received the ALDH^−^ cells. Engrafted mice were inspected once a week by visual observation and palpation for the appearance of tumours. The tumour volume (V) was determined weekly from the length (a) and the width (b) of the tumour, using the formula V = ab^2^/2. A portion of each tumour tissue was fixed in 10% formaldehyde and embedded in paraffin for IHC analysis. The frequency of tumorigenic cells (estimated with upper–lower limits) was calculated by extreme limiting dilution analysis.^16^ Mice tumour-free survivals were estimated by the Kaplan–Meier method and compared using the log-rank test.

### Clinical studies

A cohort of 121 patients with locally advanced or metastatic head-and-neck squamous cell carcinoma and treated in the palliative setting was included in a prospective study. These patients with relapsed (*n* = 90) or newly diagnosed (*n* = 31) disease were treated with chemotherapy containing cisplatin and/or fluorouracil and/or paclitaxel and cetuximab, to the discretion of the clinician. The treatment was not different between metastatic and locally advanced patients, and depended on the decision of the team in charge of the patient in each participating centre. Among the 90 relapsing patients, 58 had been treated previously by surgery, 49 had received radiotherapy, 39 concomitant radiochemotherapy and none of them had received chemotherapy alone (Supplementary Table 1A). All patients were recruited in the Aquitaine area in three University hospitals (Bergonié Institute, Saint-André Hospital and Haut-Lévêque Hospital), in different general hospital and medical centres (Libourne, Pau, Saintes, La Rochelle, Agen, Périgueux and Military Hospital Robert-Piqué, Bordeaux) and in the University hospitals of Lille and Marseille. All patients were treated in the palliative setting, for locally advanced and/or metastatic disease between February 2009 and December 2010. The study was approved by the local ethical committee, and written informed consent was provided by all the participants after a full explanation of the study was given to them. DNA was extracted from blood samples collected at baseline.

A validation study was undertaken in view of the results of the prospective study. A series of 67 consecutive patients who had given authorisation to use their biological samples for scientific studies were identified at the Cancer University Institute of Toulouse. All were suffering from relapse of head-and-neck squamous cell carcinoma and had been treated with chemotherapy. DNA was extracted from deep-frozen tumour samples since no blood sample had been kept for germline studies. However, we had already analysed in parallel in another study the presence of the polymorphism in germline and tumour DNA, and observed a 100% concordance. All patients from the validation cohort were treated in curative intent: 40 underwent surgery, 66 underwent radiotherapy (either after surgery: 39 and/or with chemotherapy: 50) and 55 received chemotherapy (in the neoadjuvant setting: 5, concomitant with radiotherapy: 50) (Supplementary Table 1B).

### In silico studies

We extended the in silico study already performed using the NCI-60 and the JFCR-45 cell line collections in order to study the association between *CYP1B1* polymorphism and drug sensitivity^1^ by using the data of the CCLE cell line collection. The CCLE collection^17^ contains 1036 cell lines of all tumour types, among which 494 have been studied pharmacologically through evaluation of the cytotoxicity of 24 anticancer drugs. Gene expression data as well as mutational profiles of a limited number of genes involved in oncogenesis are freely available on the website of the Broad Institute (http://www.broadinstitute.org/ccle/home#). In particular, data on the *CYP1B1* V432L (rs1056836) are available for 991 cell lines (494 cell lines with pharmacological data). Since 44 cell lines of the NCI collection are included in the CCLE collection, we verified that the *CYP1B1* genotypes that we had determined on the NCI collection by pyrosequencing (1) were all identical to those of the same cell lines in the CCLE database. A special attention was brought to epithelial and mesenchymal marker genes, as identified by Kohn et al.^18^ The list of the genes considered as epithelial or mesenchymal markers is provided in Supplementary Table 2.

### Statistics

When comparisons were made between two parameters, significance was estimated using Student *t* test. When three parameters were to be compared (p15, WT and VAR cells or tumours), we used ANOVA with post hoc multiple comparisons. Survival data were compared using the log-rank test.

## Results

### Characterisation of the isogenic CYP1B1-expressing cells

To characterise the phenotype associated with wild-type and variant CYP1B1 genotypes, we selected two HNSCC cell lines, CAL27 and CAL33, which do not express CYP1B1 at a detectable level as estimated by Western blot (Supplementary Fig. 1A, upper panel). This lack of protein expression was associated with a high level of methylation of the CpG islands studied in the promoter of the gene (data not shown). Lentiviral infection of both cell lines with the two polymorphic forms of CYP1B1 (wild-type V^432^ and variant L^432^) or with an empty vector (p15) generated two series of isogenic cell lines, the CYP1B1-WT, the CYP1B1-VAR and the p15 control cell lines. After DNA extraction, the exogenic CYP1B1 sequence was confirmed by pyrosequencing (Supplementary Fig. 1B). mRNAs were also extracted and retro-converted to cDNAs, and pyrosequencing was also in agreement to what was expected. In addition, CYP1B1 expression was determined by quantitative RT-PCR, which showed that the levels of expression of the two variants were similar in the two isogenic pairs (Supplementary Fig. 1C). CYP1B1 protein expression in the two types of cell lines, CYP1B1-WT and CYP1B1-VAR, was also similar (Supplementary Fig. 1A, lower panel).

**Fig. 1:**
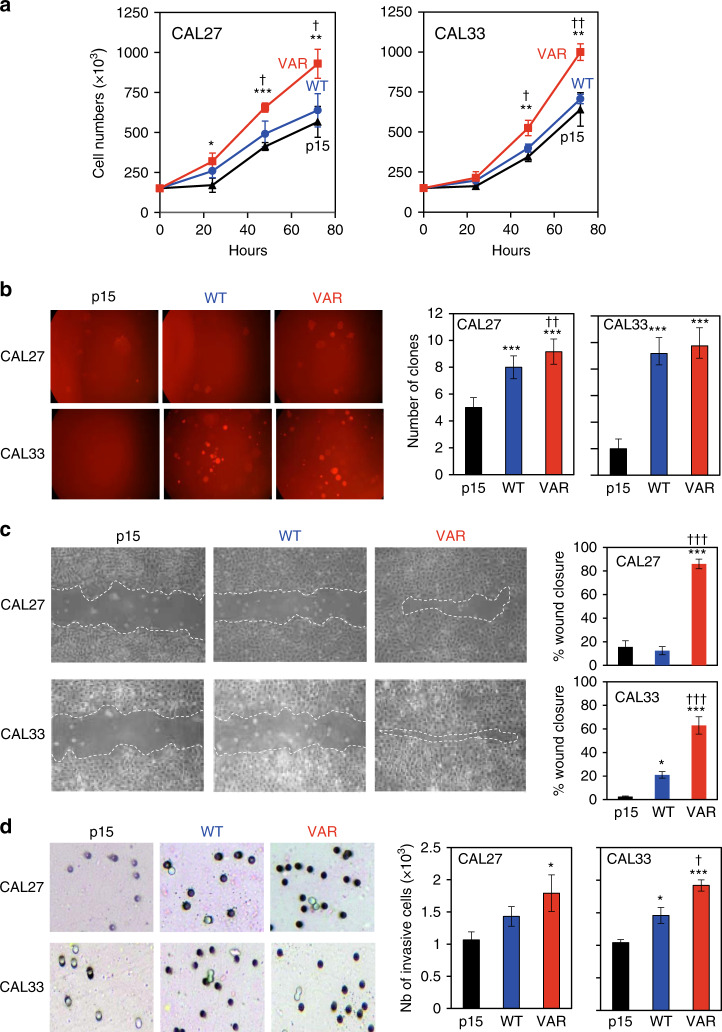
Proliferation and migration capacity of the isogenic CYP1B1 cell lines. **a** Growth curves of the CYP1B1-WT and CYP1B1-VAR cell lines as compared with control p15 in CAL27 and CAL33 cell lines. In total, 10^5^ cells were seeded on 60-mm Petri dishes and counted every 24 h for 3 days. Differences between CYP1B1-WT or CYP1B1-VAR cells and p15 cells were as follows: *p* < 0.05; **p < 0.01; ***p < 0.001. Differences between CYP1B1-WT and CYP1B1-VAR were as follows: ^†^*p* < 0.05; ^††^*p* < 0.01. **b** Cloning efficiency of the isogenic cell lines was evaluated in 24-well plates covered with 500 μl of 0.6% agar onto which 10^3^ cells suspended in 250 μl of 0.4% agar-containing culture medium were seeded. After incubation at 37 °C in a 5% CO_2_ atmosphere for 7 days, the colonies constituted by at least 50 cells were counted visually. Differences between CYP1B1-WT or CYP1B1-VAR cells and p15 cells were as follows: ***p < 0.001. Differences between CYP1B1-WT and CYP1B1-VAR were as follows: ^††^*p* < 0.01. **c** Representative scratch-test experiments performed on p15, CYP1B1-WT and CYP1B1-VAR isogenic cell lines. A scratch was made by a pipette tip 48 h after hydroxyurea treatment. Wound-healing recovery was observed 24 and 48 h after the scratch. Wound-closure quantification (% of initial split) of three independent experiments in duplicate is presented on the right (mean ± s.e.m.). Differences between CYP1B1-WT or CYP1B1-VAR cells and p15 cells were as follows: *p < 0.05; ***p < 0.001. Differences between CYP1B1-WT and CYP1B1-VAR were as follows: ^†††^*p* < 0.001. **d** Invasion assays performed on 24-well Transwell dishes. Cells (2 × 10^4^) were suspended in 200 μL of serum-free DMEM medium and placed in the top chambers; 800 μL of DMEM medium supplemented with 10% FBS was added to the bottom chambers. After removal of the non-invading cells from the top chambers, the invading cells at the bottom surface of the filter were fixed in 3% paraformaldehyde, stained and counted on the entire insert. Differences between CYP1B1-WT or CYP1B1-VAR cells and p15 cells were as follows: *p < 0.05; ***p < 0.001. Differences between CYP1B1-WT and CYP1B1-VAR were as follows: ^†^*p* < 0.05.

We then studied a series of phenotypic characteristics of the cell lines in terms of proliferation, migration, cloning efficiency, invasion capacity and chemosensitivity. Cells expressing the CYP1B1 wild-type protein (CYP1B1-WT) had a slightly higher proliferation rate than the cell lines infected with the empty vector (p15), but this difference remained non-significant over 72 h. In contrast, cells re-expressing CYP1B1 under its variant form (L^432^) (CYP1B1-VAR) had a significantly higher proliferation rate than p15 cells and CYP1B1-WT cells (Fig. 1a).

Cloning efficiency was markedly higher for CYP1B1-WT and CYP1B1-VAR cells as compared with p15 cells, especially for those derived from the CAL33 cell line (Fig. 1b). There was no difference in cloning efficiency between CAL33 CYP1B1-WT and CYP1B1-VAR cells, and only a slight difference between CAL27 CYP1B1-WT and CYP1B1-VAR cells.

Cell migration was studied after hydroxyurea-induced inhibition of cell proliferation for 48 h. We verified that proliferation was effectively blocked in these conditions by cell counting in parallel cultures (data not shown). At confluence, a scratch test was performed, and recovery was followed by photomicroscopy. Figure 1c presents representative experiments performed with the isogenic cell lines derived from both CAL27 and CAL33 cell lines. There was no significant healing in the p15 cells at 24 h, and a slight narrowing of the scratch at 48 h. In the CYP1B1-WT cells, wound healing was slightly more rapid than in p15 cells, but a highly significant difference was found between CYP1B1-WT and CYP1B1-VAR cells: in both CAL27 and CAL33 CYP1B1-VAR cells, healing was almost complete at 24 h and complete at 48 h.

A study of the invasion capacity determined with Boyden chambers also revealed differences between isogenic cell lines. For the CAL27-derived cell lines (CYP1B1-WT and CYP1B1-VAR), there was a progressive increase, from 4 h to 12 h, in cell invasion of the bottom chambers for both cell lines as compared with p15 cells, but no significant difference was found between CYP1B1-WT and CYP1B1-VAR cells (Fig. 1d). In CAL33-derived cells, a similar increase in migration capacity of CYP1B1-WT and CYP1B1-VAR cells as compared with p15 cells was detected and was significantly higher in CAL33- than in CAL27-derived cells (Fig. 1d). This can be attributed to the reintroduction of CYP1B1 and not specifically to the WT or VAR allele. Invasion capacity was maximal at 4 h after seeding; after 4 h, the filter was clogged, and the invading cells could no longer be accurately counted.

**Fig. 2:**
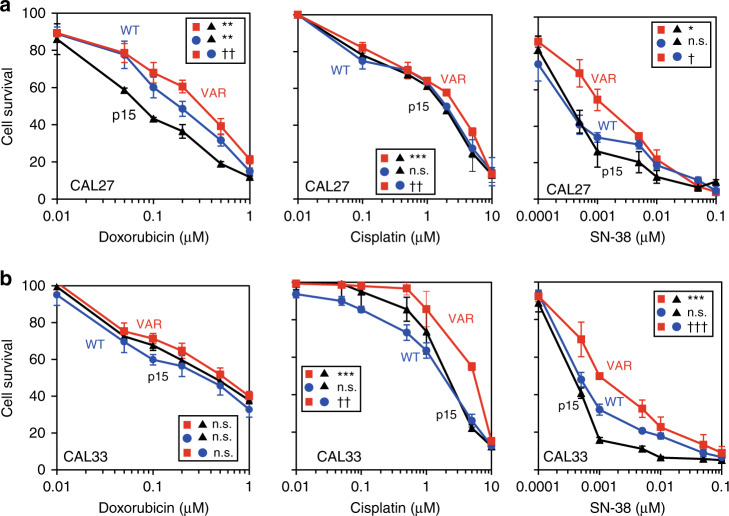
Cytotoxicity profiles of the isogenic cell lines. Cytotoxicity of cisplatin, doxorubicin and camptothecin against the isogenic cell lines p15, CYP1B1-WT (WT) and CYP1B1-VAR (VAR), was determined after 72-h incubations with various drug concentrations and cell number evaluation using the MTT assay. The results are means of at least 3 independent determinations performed in triplicate. **a** CAL27-derived cell lines; **b** CAL33-derived cell lines. IC_50_s were evaluated by interpolation and compared by ANOVA and post hoc multiple comparisons. The significance of the differences in IC_50_s is indicated in the insets as follows: *p < 0.05; **p < 0.01; ***p < 0.001. Differences between CYP1B1-WT and CYP1B1-VAR: ^†^*p* < 0.05; ^††^*p* < 0.01; ^†††^*p* < 0.001.

Chemosensitivity to a series of anticancer agents was evaluated by MTT coloration after 72-h incubation at various drug concentrations. Studying first the drugs of current use in HNSCC, we found no difference in fluorouracil cytotoxicity, but a significant difference in cisplatin cytotoxicity. As we suspected that drug mechanisms of action were of importance, we explored the activity of other drugs: an antimetabolite (gemcitabine), a spindle poison (paclitaxel) and two DNA-damaging agents, a topoisomerase II inhibitor, doxorubicin, and a topoisomerase I inhibitor, SN-38, the active metabolite of irinotecan. No significant differences were detected for fluorouracil, gemcitabine or paclitaxel (data not shown). There were no consistent differences in cytotoxicity of doxorubicin, cisplatin and SN-38 between the cell lines not expressing CYP1B1 (p15) or the WT form of the gene; however, the CAL27 CYP1B1-VAR cell line was significantly resistant to doxorubicin, cisplatin and SN-38, and the CAL33 CYP1B1-VAR cell line was significantly resistant to cisplatin and SN-38 (Fig. 2).

### In vivo characterisation of tumours originating from isogenic CYP1B1-expressing cells

**Fig. 3:**
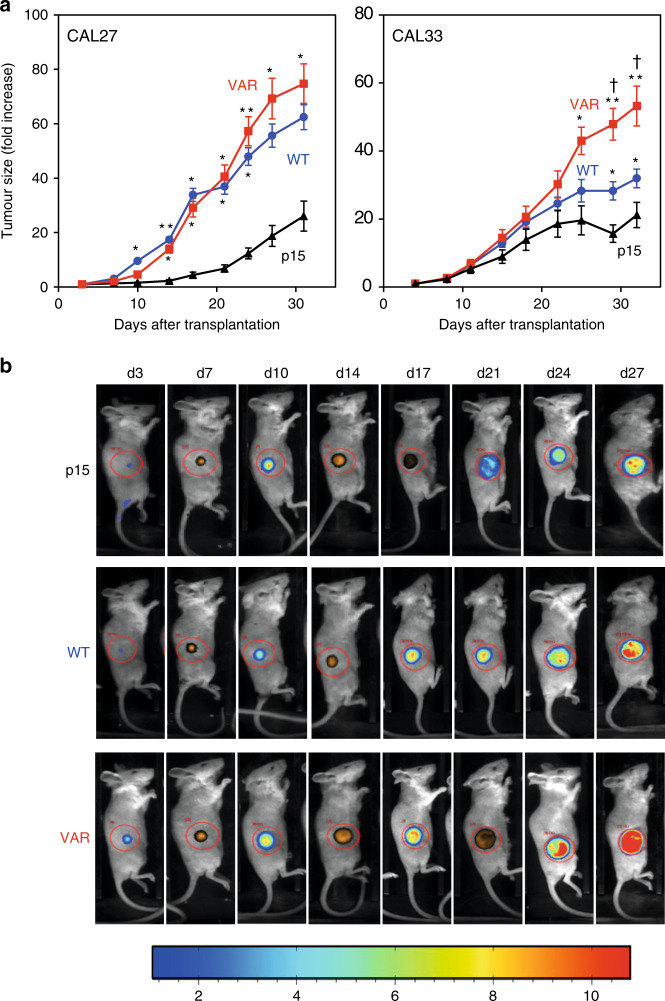
In vivo tumour growth of isogenic cell lines in immunodeficient mice. **a** In vivo tumour growth of CAL27- and CAL33-derived isogenic cell lines p15, CYP1B1-WT (WT) and CYP1B1-VAR (VAR). Groups of 5 mice were injected into their right flanks with 3 × 10^6^ tumour cells in 100 μl of medium; two independent experiments were performed, and the results were pooled. Tumour growth was evaluated by fluorescence, thanks to the expression of the tdTomato gene. Significance in tumour sizes was estimated each day using ANOVA and post hoc multiple comparisons. Differences between CYP1B1-WT or CYP1B1-VAR tumours and p15 tumours were as follows: *p < 0.05; **p < 0.01. Differences between CYP1B1-WT and CYP1B1-VAR were as follows: ^†^*p* < 0.05. **b** Tumour imaging in a representative experiment with CAL33-derived isogenic cell lines transplanted in mice. After isoflurane anaesthesia, mice were placed in a special imaging chamber of a photon bioimager (Biospace Lab, Paris, France). Signals were quantified with the M3Vision software (Biospace Lab) within defined regions of interest of identical dimensions.

**Fig. 4:**
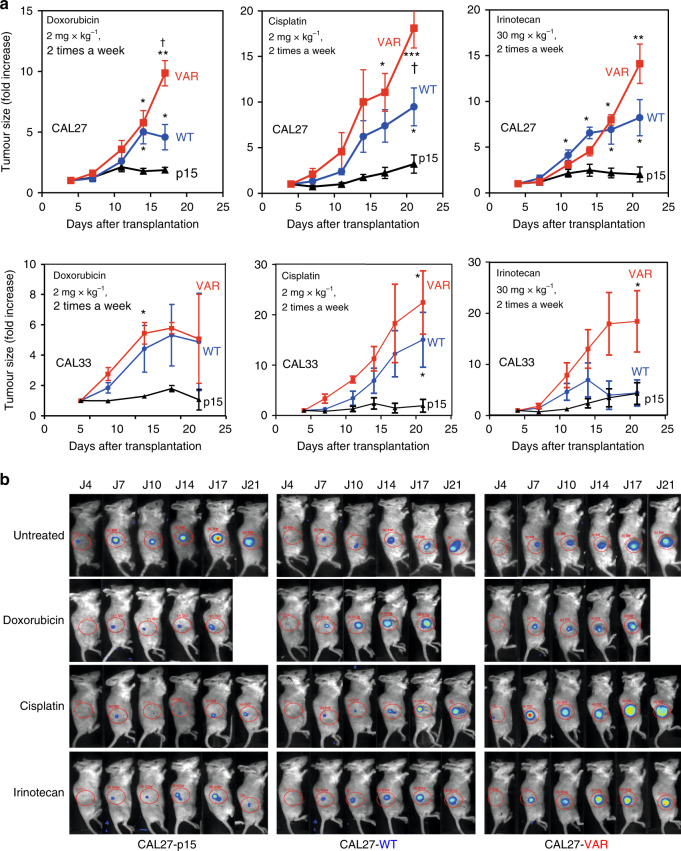
Drug-induced tumour growth inhibition of CAL27-derived isogenic cell lines in immunodeficient mice. **a** In vivo growth curves of CAL27- and CAL33-derived tumours under treatment with 3 different DNA-damaging drugs, cisplatin, doxorubicin and irinotecan. Groups of 4 mice were injected into their right flanks with 3 × 10^6^ tumour cells (p15 and CYP1B1-WT) or 1 × 10^6^ tumour cells (CYP1B1-VAR) in 100 μl of medium, because of the much faster growth of CYP1B1-VAR tumours in comparison with p15 and CYP1B1-WT tumours. Differences between CYP1B1-WT or CYP1B1-VAR tumours and p15 tumours were as follows: *p < 0.05; **p < 0.01; ***p < 0.001. Differences between CYP1B1-WT and CYP1B1-VAR: ^†^*p* < 0.05; ^††^*p* < 0.01; ^†††^*p* < 0.001. **b** Tumour imaging in selected mice for each drug tested. After isoflurane anaesthesia, mice were placed in a special imaging chamber of a photon bioimager (Biospace Lab, Paris, France) for about 20 min to acquire luminescence images. Signals were quantified with the M3Vision software (Biospace Lab) within defined regions of interest of identical dimensions.

The isogenic variants of the CAL27 and CAL33 cell lines (p15, CYP1B1-WT and CYP1B1-VAR) were grown after subcutaneous injection into the flanks of immunodeficient NOD/SCID gamma (NSG) female mice. Similar levels of expression of the transduced gene, evaluated in tumours by RT-PCR, were observed in CYP1B1-WT and CYP1B1-VAR cells of both origins. Tumour growth characteristics were strikingly different between the isogenic cells (Fig. 3). In CAL27 cells, the growth of CYP1B1-WT tumours was much more rapid than that of p15 tumours, and the difference was significant 14 days post transplantation; the growth of CYP1B1-VAR tumours was even faster, but the difference between CYP1B1-WT and CYP1B1-VAR tumours remained below significance (Fig. 4a, left panel). Similar results were obtained for the CAL33 tumours, but the difference between control and CYP1B1-expressing tumours was only significant after 25 days; a significant difference was observed between CYP1B1-WT and CYP1B1-VAR (Fig. 3a, right panel). Similar results were obtained with a control group of male mice transplanted with the CAL27 tumour cells (data not shown).

In independent experiments, CAL27 and CAL33 isogenic tumours were treated with cisplatin, doxorubicin or irinotecan (as a prodrug of SN-38) (Fig. 4). In CAL27 cells, control tumours, not expressing CYP1B1, increased by 1.5–2.5-fold under treatment, while CYP1B1-WT tumours were resistant to all three drugs, with tumour size increasing by 5–10-fold; CYP1B1-VAR tumours were resistant to a much higher degree, the average tumour increase at the end of the treatment being in the range of 10–20-fold. The difference between CYP1B1-WT and CYP1B1-VAR tumours was significant for cisplatin and doxorubicin and below the limit of significance for irinotecan. Similar results were obtained with CAL33 cells.

### Differential expression of epithelial and mesenchymal markers in CYP1B1 isogenic cells

In order to obtain insights into CYP1B1-associated features according to the polymorphic form expressed, we explored the databases of two cancer cell line collections, the NCI-60^19^ and the CCLE.^17^ We sought associations between *CYP1B1* rs1056836 genotype and gene expression profiles. Detailed results are presented as Supplementary Data. We confirmed in the CCLE collection our previous observations on the NCI-60 collection:^1^ sensitivity to the only DNA-damaging drug available in the database, topotecan, was significantly lower for the variant cell lines than for the wild-type homozygous cell lines (double-sided *t* test, *p* = 0.048, fold change = 0.773), the heterozygous cell lines presenting no significant difference in topotecan sensitivity with the variant or the wild-type homozygous cell lines. This was especially marked for the carcinoma cell lines.

Further, exploration of the gene expression profiles associated with each polymorphic form of *CYP1B1* revealed that, in both collections, variant homozygous cells for rs1056836 overexpressed a series of “epithelial” genes and underexpressed a series of “mesenchymal” genes in comparison with wild-type homozygous or heterozygous cell lines, these genes having been selected from a study by Kohn et al.^18^ Detailed results are presented in Supplementary Fig. 2A and Supplementary Table 2. As a consequence, cells expressing the Leu^432^ variant form of CYP1B1 could be considered as presenting an epithelial phenotype, whereas those expressing the Val^432^ wild-type form presented a mesenchymal phenotype.

We then asked whether the epithelial phenotype associated with the variant genotype of *CYP1B1* rs1056836 in the cell line collections was a feature also present in our isogenic cell lines. Gene expression profiles were established by RNA-seq in the isogenic CAL27 cell lines. In the first step, only the 50 genes involved in the determination of epithelial vs. mesenchymal phenotype were taken into consideration in order to reduce the limitations due to multiple testing. Overall, the mean ratios of gene expression values in the CYP1B1-VAR cells and the CYP1B1-WT cell lines were 1.55 for the 33 “epithelial genes” and 0.69 for the 17 “mesenchymal genes” (*p* = 0.0036), indicating a trend towards an epithelial phenotype in the CYP1B1-VAR cell line. Supplementary Table 3 lists the differential expression of epithelial and mesenchymal genes in the CAL27 isogenic cell lines.

In the second step, all genes were studied, and the Benjamini–Hochberg correction for multiple testing was used with a false discovery rate of 0.10. Supplementary Table 4 lists all the genes that were significantly differentially expressed between the CYP1B1-VAR and CYP1B1-WT CAL27 cell lines.

In view of these results concerning the differences in gene expression of cells expressing the wild-type or the variant form of CYP1B1, four marker proteins, representative of epithelial and mesenchymal phenotypes, were determined in the isogenic CAL27 cell lines by Western blots: ESRP1 (epithelial splicing regulatory protein 1) and RAB25 (RAS oncogene family member) for the epithelial phenotype, and GNB4 (G protein subunit beta 4) and CDH2 (N-cadherin) for the mesenchymal phenotype. These marker proteins were selected since the genes encoding them had the highest differential expression at the mRNA level. The expression of the epithelial markers was higher in the CYP1B1-VAR cell line, and that of the mesenchymal markers was in contrast higher in the CYP1B1-WT cell line, showing that the difference in mRNA expression in these cell lines was translated at the protein level (Supplementary Fig. 2B).

### Stemness features in isogenic CYP1B1-expressing cells

Since a number of the phenotypic characters that could differentiate the two CYP1B1 isogenic cell lines were related to stemness features,^20^ we explored the differential expression of stem cell cell-renewal-associated transcription factor genes in the RNA-seq results of the isogenic CAL27 cells (*POU5F1* [OCT4], *NANOG*, *KLF4* and *BMI1*, in addition to *ALDHA1*). There was a significant overexpression of *ALDHA1* in CYP1B1-VAR cells as compared with CYP1B1-WT cells (28-fold difference, *p* < 0.0037), which did not appear significant in the global analysis because of multiple-testing corrections. Similarly, *KLF4* was significantly overexpressed in this cell line (5.7-fold, *p* = 0.00085), but this was not the same for the other transcription factor genes.

**Fig. 5:**
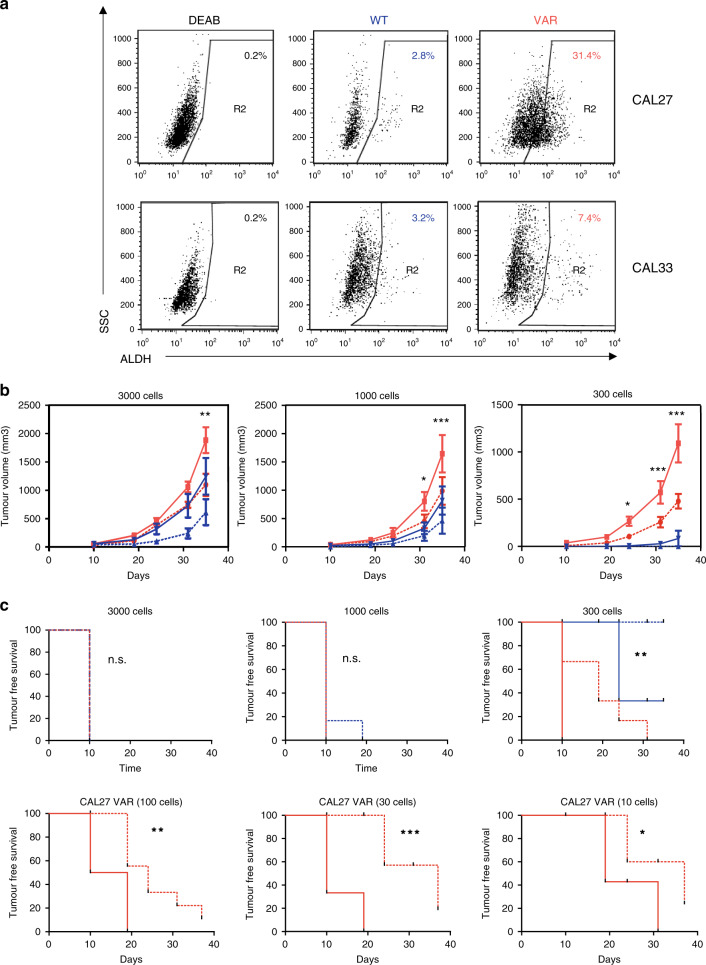
Stemness characteristics of CAL27 CYP1B1-WT and CYP1B1-VAR cells. **a** Flow cytometric analysis of CAL27- and CAL33-derived cell lines. CYP1B1-WT and CYP1B1-VAR cells were stained with ALDH reagents (Aldefluor, Stemcell Technologies Inc.). DEAB: diethylaminobenzaldehyde, as a fluorescence control. **b** Tumour progression of CAL27-derived CYP1B1-WT (blue) and CYP1B1-VAR (red) cells in NSG mice after implantation of 300–3000 ALDH^+^ (solid lines) and ALDH^−^ (dotted lines) cells. No tumour was formed when less than 300 CYP1B1-WT cells were implanted in mice. Significance between ALDH^+^ CYP1B1-VAR and CYP1B1-WT tumours was as follows: *p < 0.05; ***p < 0.001. **c** Kaplan–Meier curves of NSG tumour-free mice survival after implantation of CAL27-derived CYP1B1-WT cells (blue) or 10–3000 CYP1B1-VAR (red) cells. Implanted cell numbers were 300–3000 for CYP1B1-WT cells and 10–3000 CYP1B1-VAR cells, either ALDH^+^ (solid lines) or ALDH^−^ (dotted lines). Significance between ALDH^+^ CYP1B1-VAR and CYP1B1-WT cells for 300 implanted cells: *p* = 0.0016. Significance between ALDH^+^ and ALDH^−^ CYP1B1-VAR cells: *p < 0.05; **p < 0.01; ***p < 0.001.

In relation to the stemness character of the CYP1B1-VAR cell line, we decided to explore the content in ALDH-positive (ALDH^+^) cells in both cell lines derived from the CAL27 cell line and their ability to generate tumours in immunocompromised mice. In the exponential phase of growth, the CYP1B1-VAR cell line contained 31.4% ALDH^+^ cells, while the CYP1B1-WT cell line contained about 2.8% ALDH^+^ cells (Fig. 5a). ALDH^+^ and ALDH^−^ cells were sorted from both isogenic cell lines and injected subcutaneously at various amounts (10–3000 cells) into the flanks of groups of five 6- to 8-week-old female NSG mice; the right flank of the mouse received the ALDH^+^ cells, whereas the left flank received the ALDH^−^ cells. No tumours were formed with CYP1B1-WT cells when less than 300 cells were injected, and there were only minor differences in tumour uptake between ALDH^+^ and ALDH^−^ cells. In contrast, tumours were formed from as low as 10 injected cells in CYP1B1-VAR cells (*p* < 10^−4^ when compared with CYP1B1-WT cells) and, as expected, tumour uptake from ALDH^+^ cells was significantly higher than from ALDH^−^ cells (*p* = 0.0015). Figure 5b, c respectively presents the evolution of tumour size and the mice survival curves after injection of ALDH^+^ and ALDH^−^ cells originating either from CYP1B1-WT or CYP1B1-VAR cells.

### Different outcome of head-and-neck cancer patients according to CYP1B1 genotype

In order to determine whether the different tumour cell behaviour in cell lines and tumours bearing different CYP1B1 genotypes was translated in the clinical setting, we conducted a prospective study on 96 men and 25 women with histologically confirmed head-and-neck squamous cell carcinoma (NCT-01827956). Patients with a median age of 60.9 (range: 30.6–81.6) bearing tumours of the oral cavity (*n* = 44, 37%), oropharynx (*n* = 34, 28%), larynx (*n* = 15, 12%), hypopharynx (*n* = 20, 17%) and with nodes and/or metastases after removal of the primitive tumour (*n* = 7, 6%) were included. Overall, 78 patients had invaded nodes, and distant metastases were present in 48 patients. At the time of patient inclusion (2009–2011), HPV was not systematically researched in all participating institutions. Association studies were performed on 118 patients: 44 patients (37.3%) presented the homozygous variant genotype, 35 (29.7%) the homozygous wild-type genotype and 39 (33.0%) the heterozygous genotype. No significant deviation from the Hardy–Weinberg equilibrium was noticed.

**Fig. 6:**
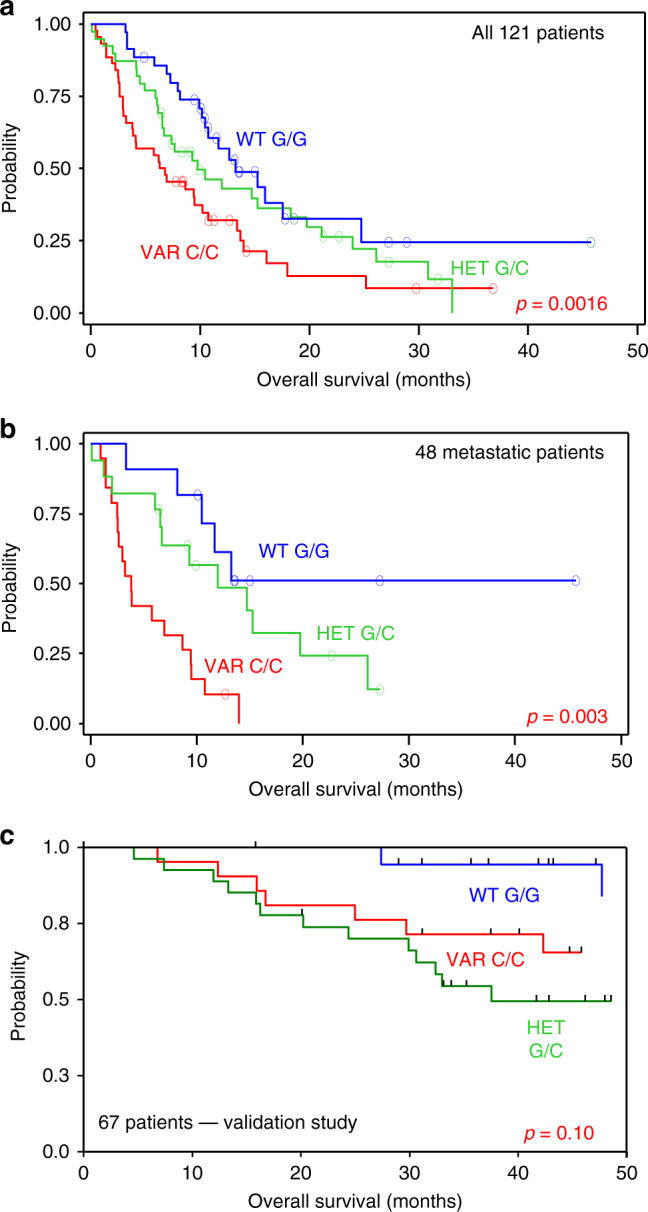
Survival curves of advanced head-and-neck cancer patients treated with chemotherapy plus cetuximab. **a** Kaplan–Meier plotting of overall survival of the 121 patients included in the study as a function of *CYP1B1* genotype. Small circles indicate censored patients. The *p* value was calculated with the log-rank test. **b** Similar representation restricted to the 48 patients who were metastatic at the time of treatment. **c** Kaplan–Meier plots of overall survival of the 67 patients of the validation study as a function of *CYP1B1* genotype. *p* = 0.10 as calculated using the log-rank test.

In the whole cohort, there was no significant association between the rate of non-progression and the genotypes. Overall survival was significantly associated with the genotypes (Fig. 6a), the wild-type patients presenting a longer median survival (13.5 months) than the variant patients (6.3 months), the heterozygous patients falling in-between (10.0 months). The log-rank test indicated a *p* value of 0.0166. Of note, 15 patients were censored at the time of analysis among those with wild-type genotype, and 9 among those with variant and with heterozygous genotypes. Event-free survival was also associated with the genotypes, but with lower significance (*p* = 0.0466) (data not shown). When studied separately, the overall survival of the metastatic population (*n* = 47) was associated with the genotypes with a high significance (*p* = 0.003), but event-free survival was not (*p* = 0.0983) (Fig. 6b). We performed a multivariate analysis of overall survival in all the patients of the cohort; TNM grading and alcohol consumption were the only prognostic factors that appeared significant.

In order to validate the clinical results, we developed a completely independent retrospective study on a series of 67 (51 men and 16 women) patients with the same pathology, but earlier in the history of the disease. All were eligible for association studies: 21 patients (31.3%) presented the CYP1B1 V432L homozygous variant genotype, 19 (28.4%) the homozygous wild-type genotype and 27 (40.3%) the heterozygous genotype. No significant deviation from the Hardy–Weinberg equilibrium was noticed. No patient with homozygous wild-type genotype developed local relapse, while 6 and 8 patients bearing, respectively, homozygous variant and heterozygous genotypes, developed local relapses. The overall median survival was 64.8 months. The homozygous wild-type patients had a percent survival at 3 years of 94.4%, while the homozygous variant and heterozygous patients had, respectively, percent survivals at 3 years of 71.4 and 54.4% (*p* = 0.104) (Fig. 6c). Regrouping homozygous variants and heterozygous patients gave a *p* value of 0.07. Despite the fact that the significance level of 0.05 was not reached for the second cohort, there is a common trend associating the CYP1B1 polymorphism to patients’ survival.

## Discussion

CYP1B1 has been recognised for a long time as an unusual cytochrome P450. As most cytochromes’ P450 of the 1, 2 and 3 families, this protein is responsible for various metabolic alterations of drugs and xenobiotics; its recognised substrates are polycyclic aromatic hydrocarbons and 17β-oestradiol. However, it has been shown that it is mostly expressed in tumours rather than in normal tissues, and has been presented as a “universal tumour marker”^9^ that should be prioritised for therapeutic targeting.^10^ Mutations of this gene have been associated with congenital glaucoma,^21^ and the V432L polymorphism (rs1056836) has been associated with a series of pathological factors that cannot be all mentioned here. Just to name a few, the C-allele carriers had a significantly higher risk of laryngeal cancer than the G-allele carriers;^22^ they also had shorter survival parameters after taxane treatment for breast^23^ and prostate^5^ cancers. Some authors noticed a slight difference in catalytic activity towards oestradiol, 17β-oestradiol or benzo[a]pyrene,^24,25^ while others did not find any significant difference in catalytic activity between the two polymorphic forms of CYP1B1.^26^

It may appear unusual that a SNP conserving the hydrophobic nature of the amino acid change, and in a region far from the catalytic domain of the protein, could be associated with important phenotypic variations such as those mentioned in the literature; it may be argued that this SNP might be in strong linkage disequilibrium with another SNP involved in gene expression regulation. This is the reason why we generated isogenic cell lines, differing only by the presence of this single variation in the informative sequence, and expressing the resulting protein at comparable levels; using this model, we demonstrated that the amino acid change was by itself responsible for the alterations of important phenotypic properties. The 3D structure of the protein, as extracted from the UniProt database (http://www.ebi.ac.uk/pdbe/entry/pdb/3pm0/analysis) from the data of Wang et al.^27^ clearly shows that the amino acid at position 432 is located at the periphery of the protein (Supplementary Fig. 3) and may be, therefore, quite accessible for protein–protein interactions; a difference in the partners of CYP1B1 as a function of the presence of a valine or a leucine may thus explain the different phenotypes presented by the two forms of the protein. The fact that the variation is distant from the enzyme-active centre would also explain why it induces no major difference in catalytic activity.

Thanks to the generation of isogenic cell lines and the study of their properties, we first confirmed the importance of the 432 position in CYP1B1. The study of the literature on this variation is relatively difficult, and several sources of confusion may be encountered: (1) this is a G → C transversion, resulting on the other strand in a C→G transversion; (2) the rs alleles are reported in the NCBI dbSNP database in reverse orientation with respect to the conventional chromosome sequence; (3) the two variations are of close allelic frequencies, and the “wild-type” in a study may well be the “variant” in another study. We consider here that the G in the genome, responsible for the Val^432^ amino acid, is the “wild-type” and the C, responsible for the Leu^432^ in the protein sequence, is the “variant”. A study of the publications mentioned in the reference list led us to the conclusion that this is the Leu^432^ that is found related to the pejorative features associated with the polymorphism (increase in cancer risk, poor disease prognosis and resistance to therapy).

In our study, the expression of CYP1B1 by itself does not seem to be predominantly involved in cell proliferation, tumour growth, cell motility or acquisition of an epithelial or mesenchymal phenotype: the cells infected with the wild-type form of the CYP1B1 cDNA present only slight differences with those infected with the empty virus (which have no detectable CYP1B1 expression). When evaluating drug sensitivity, the in vitro results concerning three drugs, cisplatin, doxorubicin and SN-38, did not differ between cells expressing no CYP1B1 and those expressing the wild-type CYP1B1, except for doxorubicin in CAL27 cells. We have already noticed that CYP1B1 expression is not related to drug sensitivity in the NCI-60 panel;^1^ CYP1B1 expression is associated with topotecan resistance in the CCLE panel, but no data are available on doxorubicin or cisplatin cytotoxicity. In contrast, the tumours derived from our isogenic cell lines presented a significant resistance to the three drugs when they re-expressed CYP1B1, which may result from specific alterations of endogenous molecules by CYP1B1, such as oestrogens, the principal CYP1B1 substrate. However, no difference was seen according to the gender of the mice.

In a recent article published during the course of our experimentation, Kwon et al.^28^ have shown important effects of overexpression and knockdown of CYP1B1 in a model of breast cancer cells; they observed that CYP1B1 induces cell proliferation and metastasis, and enhances cell invasion. It is remarkable that the MCF7 and the MDA-MB-231 cell lines that were used by these authors to assess the CYP1B1 properties are both variant homozygous cell lines, so that our results are in agreement with their conclusions on the relationship between the level of CYP1B1 expression and the phenotypic properties of cell proliferation and migration. We think that the important effects they observe upon inducing or knocking down CYP1B1 expression are mainly due to the fact that the variant protein is the only one to be expressed in these cell lines, as in our CYP1B1-VAR cell lines, originating from CAL27 or CAL33 cell lines not expressing CYP1B1 at the basal state.

The CYP1B1 V432L polymorphism has a much more drastic effect on cell phenotype than the level of expression of CYP1B1 by itself: the cells infected with the variant form of CYP1B1 strongly differ from the other isogenic cells, in terms of cell proliferation, motility, invasiveness and chemosensitivity, and of tumour growth and drug sensitivity after in vivo transplantation. They especially exhibit a high degree of resistance to cisplatin, both in vitro and in vivo. They are also characterised by the acquisition of epithelial markers and the loss of mesenchymal markers. This is in agreement with the fact that, in the cell line collections we have explored, the CYP1B1 variant genotype is associated with an epithelial phenotype (75% of the epithelial cell lines bear the homozygous variant genotype in the NCI-60 collection). However, this appears contradictory with the fact that the CAL27 and CAL33 CYP1B1-VAR cells display phenotypic properties generally associated with the epithelial-to-mesenchymal transition, especially their enhanced motility and invasiveness.^20^ This distortion underlines the complexity of the transcriptional regulation of the epithelial-to-mesenchymal transition.

The CAL27 cell line expressing the variant CYP1B1 genotype displays marked stemness properties as compared with the wild-type cell line. This was assessed by the higher proportion of ALDH-expressing cells and, more important, by the much higher tumour uptake and growth rate of these cells when they display the variant genotype. This is in agreement with their enhanced motility and invasiveness, as well as with their chemoresistance. However, the distortion between the dominance of epithelial characters of L^432^ CYP1B1 variant over the mesenchymal characters and the presence of stemness markers and properties remains puzzling, as mentioned above. It should be recalled that the epithelial-to-mesenchymal transition is a dynamic process, so that the epithelial characters of cultured cells not yet engaged in dissemination do not preclude their ability to spread and form metastases as expected from stem cells. The research of epithelial vs. mesenchymal markers in our isogenic cells after implantation in immunocompromised mice could bring an answer to this question.

The in vitro and in vivo phenotypic properties associated with the polymorphism have a clear translation in the clinics. We had already shown that the variant genotype was associated with lower response rates of patients treated for breast cancer in the neoadjuvant setting with the FEC combination (fluorouracil, epirubicin, cyclophosphamide), which was not observed for patients treated with a taxane–epirubicin combination.^6^ We show here, in a prospective clinical study dedicated to pharmacogenetics, that the overall survival of patients with advanced head-and-neck cancer could also be dependent upon CYP1B1 polymorphism. This was especially significant for the subgroup of metastatic patients, which showed that the V432L polymorphism of CYP1B1 may interfere with fundamental features of cancer cell fate orientation. The validation study failed to reach significance (*p* = 0.07), probably because of an insufficient number of patients; however, the trend is the same as in the prospective study that had included twofold more patients, and we can consider the validation study as positive.

In conclusion, we have demonstrated for the first time the crucial importance of a frequent CYP1B1 polymorphism for cancer cell proliferation, migration and resistance to chemotherapy, both in vitro and in vivo. This polymorphism also strongly influences head-and-neck cancer patients’ survival. Further studies oriented towards patients’ CYP1B1 genetic differences may prove useful for the understanding of the metastatic process, as well as for patients’ prognosis and prediction of response to treatment. It must be underlined that the CYP1B1 variation studied is a polymorphism present both in patients’ germline and tumour DNA. As other polymorphisms,^29^ it may have an effect on metastasis facilitation or inhibition.

## Supplementary information

Supplementary material

## Data Availability

Raw results of the RNA-seq are available on the Annotare platform (ArrayExpress accession #E-MTAB-8512).
