# Supplementary material for: Cytochrome P450 1B1 polymorphism drives cancer cell stemness and patient outcome in head-and-neck carcinoma
Source: Br J Cancer. 2020 Jun 22;123(5):772–84. doi: 10.1038/s41416-020-0932-5 (PMC7462978; doi:10.1038/s41416-020-0932-5)

Supplemental data (Supplemental Tables 1–4 and Supplemental Figures 1-3)

1. Validation of the model

Supplemental Figure 1 gathers the validation data obtained on the isogenic cell lines by Western blots, pyrosequencing and RT-PCR

Supplemental Figure 1. Validation of the experimental model

A. Levels of expression of CYP1B1 determined by western blotting in a series of cancer cell lines: SW620 (colon cancer); DU145 (prostate cancer); MCF7 (breast cancer); SW780 (bladder cancer); and in the original CAL27 and CAL33 cell lines and in the corresponding transduced cell lines, CYP1B1-WT and CYP1B1-VAR.

B. Pyrosequencing of the wild-type human CYP1B1 cDNA (1697G) after transduction with the lentiviral pER51CYP1B1-WT or pER51CYP1B1-VAR obtained by site-directed mutagenesis.

C. qRT-PCR of the CYP1B1 mRNA extracted from the isogenic cell lines transduced with the lentiviral vectors pER51 (p15, control), pER51CYP1B1-WT (WT) or pER51CYP1B1-VAR (VAR). MK: size marker (100 bp ladder).

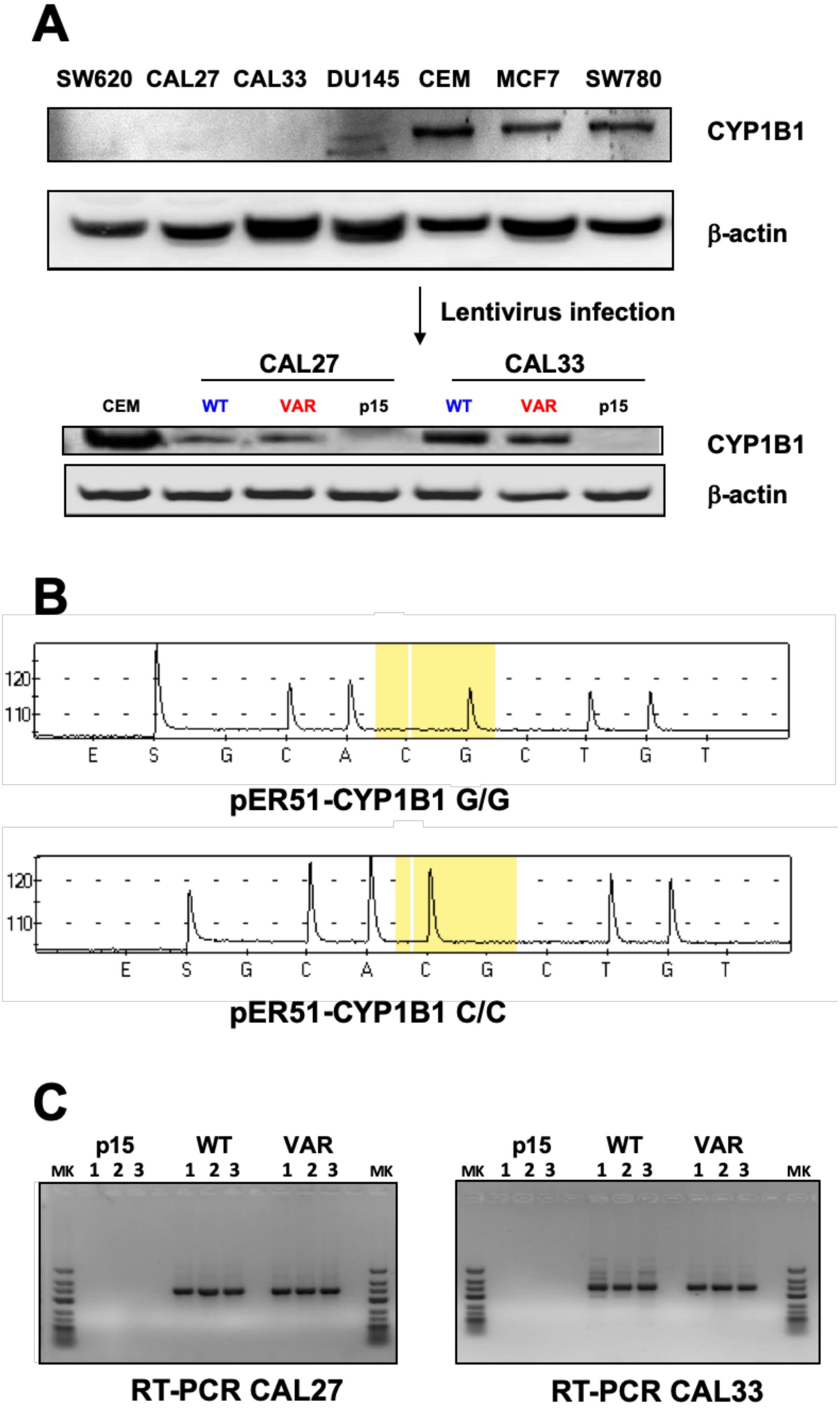
A
SW620 CAL27 CAL33 DU145 CEM MCF7 SW780
CYP1B1
β-actin
Lentivirus infection
CAL27
CAL33
CEM WT VAR p15 WT VAR p15
CYP1B1
β-actin
B
120
110
E S G C A C G C T G T
pER51-CYP1B1 G/G
120
110
E S G C A C G C T G T
pER51-CYP1B1 C/C
C
p15 WT VAR
MK 1 2 3 1 2 3 1 2 3 MK
RT-PCR CAL27
p15 WT VAR
MK 1 2 3 1 2 3 1 2 3 MK
RT-PCR CAL33

2. Clinical studies

Supplemental Table 1A. Characteristics of the patients of the prospective cohort

		Number	p. cent
Gender	Male	96	79.3
	Female	25	20.7
Age	Mean ± SD	60.5 ± 10.6	
	Min – Max	30.6 – 81.6	
Primary localization	Oral cavity	44	36.4
	Oropharynx	35	28.9
	Larynx	15	12.4
	Hypopharynx	20	16.5
	Primitive removed	7	5.8
Relapsing	No	31	25.6
	Yes	90	74.4
Performance status at inclusion	0	17	14.0
	1	75	62.0
	2	28	23.1
	3	1	0.8
Previous treatment (relapsing patients)*	Surgery	58	64.4
	Radiotherapy	49	54.4
	Chemotherapy	39	43.3
TNM grading at inclusion	T1	9	7.4
	T2	22	18.2
	T3	19	15.7
	T4	57	47.1
	Tx	14	11.6
	N0	39	32.2
	N1	12	9.9
	N2a	6	5.0
	N2b	22	18.2
	N2c	25	20.7
	N3	10	8.3
	Nx	7	5.8
	M0	100	82.6
	M1	16	13.2
	Mx	5	4.1
Status at inclusion	Locoregional relapse (LR)	58	47.9
	Locoregional primary (LP)	15	12.4
	Metastatic relapse	30	24.8
	LR + metastasis	16	13.2
	LP + metastasis	2	1.7
Chemotherapy*	Cetuximab	121	100
	Platinum	110	90.9
	Fluorouracil	71	59.1
	Paclitaxel or docetaxel	17	13.6
	Methotrexate	5	4.5

* Totals are higher than 121 and percentages are higher than 100 because of combined treatments

Supplemental Table 1B. Characteristics of the patients of the retrospective cohort

		Number	p. cent
Gender	Male	51	76.1
	Female	16	23.9
Age	Mean ± SD	59.4 ± 9.1	
	Min – Max	42.3 – 84.9	
Performance status	0	41	61.2
	1	24	35.8
	2	2	3.0
Tobacco use	Present smoker	39	58.2
	Former smoker	16	23.9
	Never smoker	12	17.9
Alcoholism	Yes	30	44.8
	Weaned	10	14.9
	No	27	40.3
p16 status	Negative	39	58.2
	Positive	28	41.8
Previous cancer	No	58	86.6
	Yes	9	13.4
TNM grading	T1	6	9.0
	T2	25	37.3
	T3	12	17.9
	T4	24	35.8
	N0	8	11.9
	N1	9	13.4
	N2a	1	1.5
	N2b	34	50.7
	N2c	10	14.9
	N3	5	7.5
	M0	14	20.9
	M1	53	79.1
Chemotherapy	Neo-adjuvant	5	7.5
	Concomitant, with platinum	50	74.6
	Concomitant, w/o platinum	12	17.9
Surgery	No	27	40.3
	Yes	40	59.7
Radiotherapy	No	1	1.5
	Yes, after surgery	39	58.2
	Yes, w/o surgery	27	40.3

Supplemental Table 2. Differential expression of epithelial and mesenchymal genes in the NCI-60 and the CCLE cell line collections.

Epithelial genes					Mesenchymal genes				
	Differential expression (NCI-60)		Differential expression (CCLE)			Differential expression (NCI-60)		Differential expression (CCLE)	
	Fold change	*p* value	Fold change	*p* value		Fold change	*p* value	Fold change	*p* value
CLDN7	8.87	$8.09.10^{-5}$	2.75	$6.97.10^{-4}$	*LIX1L*	0.113	$9.30.10^{-5}$	0.850	$2.94.10^{-1}$
ESRP2	8.11	$1.93.10^{-4}$	2.01	$7.98.10^{-3}$	*CCDC88A*	0.142	$4.17.10^{-4}$	0.748	$5.55.10^{-2}$
CLDN4	7.95	$1.25.10^{-4}$	1.54	$3.00.10^{-3}$	*CMTM3*	0.158	$1.21.10^{-3}$	0.724	$6.54.10^{-3}$
EPN3	7.69	$1.56.10^{-4}$	1.52	$6.08.10^{-2}$	*VIM*	0.204	$7.21.10^{-3}$	0.368	$2.41.10^{-3}$
RAB25	7.13	$5.96.10^{-4}$	2.94	$7.61.10^{-3}$	*EMP3*	0.238	$1.58.10^{-2}$	0.503	$4.08.10^{-2}$
GRHL2	7.08	$2.88.10^{-4}$	2.26	$1.86.10^{-3}$	*MSN*	0.251	$1.09.10^{-2}$	1.146	$6.38.10^{-1}$
OVOL1	7.02	$2.85.10^{-4}$	1.44	$6.51.10^{-3}$	*IKBIP*	0.253	$1.04.10^{-2}$	0.816	$2.07.10^{-1}$
ANXA9	6.38	$8.85.10^{-4}$	1.48	$4.82.10^{-2}$	*GNB4*	0.277	$2.68.10^{-2}$	0.436	$2.56.10^{-5}$
EPHA1	6.38	$7.69.10^{-4}$	1.23	$6.12.10^{-3}$	*BICD2*	0.283	$2.31.10^{-2}$	0.954	$5.26.10^{-1}$
MARVELD3	6.25	$8.17.10^{-4}$	1.79	$6.53.10^{-3}$	*QKI*	0.315	$3.18.10^{-2}$	0.906	$4.67.10^{-1}$
S100A14	6.22	$1.54.10^{-3}$	2.02	$3.16.10^{-2}$	*STARD9*	0.32	$3.74.10^{-2}$	0.828	$1.02.10^{-1}$
TJP3	6.11	$1.22.10^{-3}$	1.32	$1.16.10^{-1}$	*AP1M1*	0.322	$5.25.10^{-2}$	0.851	$9.70.10^{-2}$
GRHL1	6.06	$9.08.10^{-4}$	1.37	$8.41.10^{-2}$	*CDH2*	0.405	$1.12.10^{-1}$	0.703	$2.01.10^{-1}$
CLDN3	5.95	$2.72.10^{-3}$	1.22	$4.52.10^{-1}$	*ZEB1*	0.44	$1.50.10^{-1}$	0.539	$3.66.10^{-3}$
ATP2C2	5.80	$1.05.10^{-3}$	1.15	$1.74.10^{-1}$	*ZEB2*	0.518	$2.51.10^{-1}$	0.676	$2.25.10^{-2}$
EPCAM	5.72	$2.59.10^{-3}$	4.04	$6.59.10^{-3}$	*SNAI2*	0.601	$2.47.10^{-1}$	0.695	$3.33.10^{-1}$
ST14	5.51	$3.31.10^{-3}$	1.81	$2.23.10^{-3}$	*SNAI1*	2.015	$2.21.10^{-1}$	0.839	$5.08.10^{-2}$
MYO5B	5.43	$1.62.10^{-3}$	1.70	$9.74.10^{-4}$					
CRB3	5.20	$3.01.10^{-3}$	1.41	$9.68.10^{-3}$					
AP1M2	5.12	$2.29.10^{-3}$	1.63	$3.39.10^{-2}$					
CGN	5.01	$3.05.10^{-3}$	1.52	$3.30.10^{-2}$					
IRF6	5.00	$4.65.10^{-3}$	1.70	$1.29.10^{-2}$					
MARVELD2	4.52	$8.50.10^{-3}$	1.96	$9.21.10^{-3}$					
ESRP1	4.47	$7.03.10^{-3}$	2.45	$2.50.10^{-2}$					
PPL	4.41	$7.47.10^{-3}$	1.93	$1.77.10^{-2}$					
PCDH1	4.35	$7.37.10^{-3}$	1.13	$8.62.10^{-2}$					
LNX1	4.24	$4.50.10^{-3}$	0.91	$4.30.10^{-1}$					
LLGL2	4.23	$1.21.10^{-2}$	1.41	$2.84.10^{-2}$					
CAMSAP3	4.06	$1.31.10^{-2}$	1.19	$1.43.10^{-1}$					
EHF	3.75	$1.60.10^{-2}$	2.46	$1.18.10^{-3}$					
CDH1	3.36	$3.44.10^{-2}$	3.14	$1.64.10^{-3}$					
ADAP1	3.35	$3.65.10^{-2}$	1.33	$3.16.10^{-2}$					
PRSS8	11.84	$7.42.10^{-6}$	1.81	$1.84.10^{-2}$					

Fold change is the ratio of the average of gene expression in the CYP1B1 rs1056836 variant cell lines to the average of gene expression in the CYP1B1 wild-type + heterozygous cell lines. *p* values were estimated by the Student's *t*-test.

Supplemental Table 3. Epithelial, mesenchymal and stemness markers gene expression ratios in CAL27–derived CYP1B1-WT and CYP1B1-VAR cell lines as evaluated by RNAseq.

Epithelial genes differential expression			Mesenchymal genes differential expression		
	Fold change	*p* value		Fold change	*p* value
S100A14	5.95	4.00×10^{-5}	*VIM*	0.031	2.58×10^{-3}
ATP2C2	3.40	1.47×10^{-2}	*ZEB1*	0.169	1.00×10^{-1}
ESRP1	3.31	7.35×10^{-4}	*GNB4*	0.250	3.01×10^{-3}
GRHL1	2.53	7.87×10^{-3}	*STARD9*	0.277	3.39×10^{-2}
RAB25	2.51	4.93×10^{-3}	*ZEB2*	0.299	3.15×10^{-2}
PPL	2.49	1.40×10^{-2}	*CDH2*	0.357	1.63×10^{-2}
ST14	2.35	6.37×10^{-3}	*AP1M1*	0.660	4.58×10^{-2}
TJP3	1.86	2.75×10^{-2}	*CMTM3*	0.679	4.63×10^{-2}
EPN3	1.81	3.05×10^{-2}	*BICD2*	0.709	4.53×10^{-2}
LLGL2	1.70	3.84×10^{-2}	*SNAI2*	0.741	6.00×10^{-2}
CLDN4	1.52	3.57×10^{-2}	*LIX1L*	0.773	$6.96.10^{-2}$
ADAP1	1.46	4.73×10^{-2}	*EMP3*	0.961	9.75×10^{-2}
CAMSAP3	1.45	5.92×10^{-2}	*IKBIP*	1.005	9.93×10^{-2}
GRHL2	1.42	4.43×10^{-2}	*QKI*	1.067	8.99×10^{-2}
PCDH1	1.40	4.67×10^{-2}	*SNAI1*	1.098	9.21×10^{-2}
ANXA9	1.33	8.03×10^{-2}	*CCDC88A*	1.315	6.08×10^{-2}
CGN	1.23	6.78×10^{-2}	*MSN*	1.364	4.89×10^{-2}
EPHA1	1.19	7.50×10^{-2}			
MARVELD2	1.16	7.88×10^{-2}			
MYO5B	1.16	8.17×10^{-2}	Stemness markers differential expression		
ESRP2	1.12	8.66×10^{-2}	*POU5F1*	0.784	8.7×10^{-1}
MARVELD3	1.12	8.44×10^{-2}	*NANOG*	0.032	1.3×10^{-1}
AP1M2	1.01	9.84×10^{-2}	*KLF4*	5.715	8.5×10^{-4}
IRF6	1.00	9.97×10^{-2}	*BMI1*	1.622	4.2×10^{-1}
CRB3	0.91	9.32×10^{-2}	*ALDH1A1*	27.85	3.7×10^{-3}
EPCAM	0.87	7.62×10^{-2}			
PRSS8	0.84	7.04×10^{-2}			
OVOL1	0.82	7.99×10^{-2}			
CLDN7	0.64	$4.25.10^{-2}$			
CDH1	0.59	2.50×10^{-2}			
LNX1	0.40	5.50×10^{-2}			
EHF	0.28	1.43×10^{-3}			
CLDN3	0.22	3.33×10^{-2}			

Supplemental Table 4. Genes significantly differentially expressed in CAL27–derived CYP1B1-WT and CYP1B1-VAR cell lines.

Gene	Gene function	Ratio VAR/WT	Raw *p* value	Corrected *p* value
H19, MIR675	Imprinted maternally expressed transcript (non-protein coding)	93.3	5×10^{-5}	0.0165
EEF1A2	Eukaryotic translation elongation factor 1 alpha 2	31.5	5×10^{-5}	0.0165
AQP3	Aquaporin 3 (Gill blood group)	29.1	5×10^{-5}	0.0165
FGF2	Fibroblast growth factor 2	15.4	1.5×10^{-4}	0.0421
OASL	2'-5'-oligoadenylate synthetase-like	13.1	5×10^{-5}	0.0165
ZC3HAV1	Zinc finger CCCH-type protein, antiviral 1	7.0	1.5×10^{-4}	0.0421
KYNU	Kynureninase	0.0937	5×10^{-5}	0.0165
MX1	MX dynamin-like GTPase 1	0.0883	5×10^{-5}	0.0165
IFI6	Interferon alpha-inducible protein 6	0.0663	5×10^{-5}	0.0165
IGFBP3	Insulin-like growth factor binding protein 3	0.0602	5×10^{-5}	0.0165
SERPINA3	Serpin peptidase inhibitor, clade A member 3	0.0385	1×10^{-4}	0.0304
TNC	Tenascin C	0.0305	5×10^{-5}	0.0165
CCL2	Chemokine (C-C motif) ligand 2	0.0247	5×10^{-5}	0.0165
CD74	CD74 molecule (associates to the class II major histocompatibility complex)	0.0201	5×10^{-5}	0.0165
IKZF1	IKAROS family zinc finger 1 protein	0.0026	5×10^{-5}	0.0165

We have excluded from this list the genes whose expression was undetectable in one of the cell lines and below the limit of 10 units in the other one.

3. *In silico* study

Two features could be studied in the cancer cell line collections of the NCI-60 and the CCLE in relation to our experimental observations: the associations between *CYP1B1* genotype and (1) sensitivity to anticancer drugs; and (2) epithelial / mesenchymal phenotype. We have already published the association of *CYP1B1* genotype with cell chemosensitivity in the NCI-60 panel (1); in the CCLE collection, among the 24 drugs whose cytotoxicity was determined, most of them concern targeted therapies, and few cytotoxic drugs have been studied; especially, no data on doxorubicin or cisplatin are available. The only drug relevant to our *in vitro* and *in vivo* studies is topotecan, a potent topoisomerase I inhibitor. Analysis of the relationship between drug toxicity and *CYP1B1* expression and polymorphism revealed two significant associations: the coefficient of correlation between *CYP1B1* expression (log-converted) and topotecan IC_{50} (log-converted) was –0.210 (ddf=492, p=2×10^{-6}) indicating that high *CYP1B1* mRNA expression is significantly associated with drug resistance; and the cytotoxicity of topotecan was significantly lower against the variant cell lines (C/C genotype) than against the wild-type homozygous cell lines (G/G genotype) (double-sided t-test, p=0.048, fold-change=0.773), the heterozygous C/G cell lines presenting no significant difference in topotecan sensitivity with the C/C or the G/G cell lines. It was possible to separate the CCLE cell lines in two categories: carcinoma (303 lines) and non-carcinoma (187 lines). It is worthy to note that the significant association between topotecan cytotoxicity and CYP1B1 genotype was prominent in the carcinoma cell lines (p=0.0056) but not significant in the non-carcinoma cell lines (p=0.273).

We then identified the gene expression patterns associated with the *CYP1B1* rs1056836 genotype in the NCI-60 and the CCLE panels. When looking for the genes whose expression was higher in the cell lines with variant homozygous genotype than in the other cell lines, it appears that, in the NCI-60 collection, all 33 genes identified as 'epithelial' in Kohn's study[18] were significantly overexpressed (fold change = 11.8 to 3.35, p=7×10^{-6} and 4×10^{-2}); similarly, in the CCLE collection, 25 out of these 33 genes were significantly overexpressed in the *CYP1B1* variant cell lines (fold change = 3.21 to 1.23, p=7×10^{-4} to 5×10^{-2}) (Supplemental Table 2). Conversely, in the NCI-60 collection, 11 out of 17 genes identified as 'mesenchymal' in Kohn's study[18] were significantly under-expressed (fold change = 0.113 to 0.320, p=9×10^{-5} to 4×10^{-2}), and in the CCLE collection, 7 out of these 17 genes were also under-expressed (fold change=0.368 to 0.932, p=2.6×10^{-5} to 5×10^{-2}) (Supplemental Table 2). A heat map built on the wild-type and variant CYP1B1 cell lines of the NCI-60 (Supplemental Figure 1A) shows that the cell lines with CYP1B1 variant genotype are uniformly distributed in the epithelial and mesenchymal clusters while the cell lines with CYP1B1 wild-type or heterozygous genotypes segregate in the mesenchymal cluster

(17 out of 18) (*p*=0.003). The expression of the epithelial markers was higher in the CYP1B1-VAR cell line and that of the mesenchymal markers were in contrast higher in the CYP1B1-WT cell line, showing that the difference in mRNA expression in these cell lines was translated at the protein level (Supplemental Figure 1B). It should be mentioned in addition that the epithelial cell lines are significantly resistant to cisplatin (*p*=0.0056) and camptothecin (*p*=0.012) and marginally resistant to doxorubicin (*p*=0.173).

Gene expression profiling of the CAL27 isogenic cell lines

The expression of the 33 epithelial and the 17 mesenchymal genes in the CAL27 isogenic cell lines (*CYP1B1* rs1056836 wild-type and variant) is presented in Supplemental Table 3. Only these 50 genes were explored in the first step of the analysis.

In the second step, all genes were taken in consideration. The expression of the genes that are significantly differentially expressed between CYP1B1-WT and CYP1B1-VAR CAL27 cell lines after Benjamini-Hochberg correction for multiple testing is presented in Supplemental Table 4.

Supplemental Figure 2. Differential expression of epithelial and mesenchymal markers according to CYP1B1 V432L genotype

A. Clustered image map of NCI-60 mRNA expression levels for epithelial and mesenchymal genes. The genes included in clustering are the epithelial and mesenchymal genes listed in Supplemental Table 1. This allows to discriminate between epithelial cell lines (on the right) and mesenchymal cell lines (on the left). The epithelial cluster contains 9 cell lines with CYP1B1 variant genotype, two heterozygous (HT) cell lines and 1 with CYP1B1 wild-type genotype. The mesenchymal cluster contains 11 C/C cell lines, 20 HT and 17 WT cell lines. The difference of CYP1B1 genotype distribution between the two clusters is significant (*p*=0.003).

B. Representative Western blots of epithelial and mesenchymal protein markers. Four protein markers, representative of epithelial and mesenchymal phenotypes, were determined by Western blots of CAL27 cells: ESRP1 (epithelial splicing regulatory protein 1) and RAB25 (RAS oncogene family member) for the epithelial phenotype and GNB4 (G protein subunit beta 4) and CDH2 (N-cadherin) for the mesenchymal phenotype. Thirty micrograms of proteins were separated by 10% sodium dodecyl sulfate–polyacrylamide gel electrophoresis and transferred onto nitrocellulose membranes, which were then incubated with primary antibodies against ESRP1, RAB25, GNB4, CDH2 (N-cadherin) (all diluted at 1:500, Atlas antibodies, Ozyme) and peroxidase-conjugated secondary anti-rabbit or anti-mouse antibodies. GAPDH (Millipore antibody, diluted at 1:50,000)

was used as a protein-loading reference control in each cell line. Signals were detected using the Enhanced Chemiluminescence Reagent (Millipore). Columns represent mean ratios ± s.d. (protein signal / GAPDH signal). Differences between CYP1B1-WT and CYP1B1-VAR cells as follows: † $p<0.05$; †† $p<0.01$.

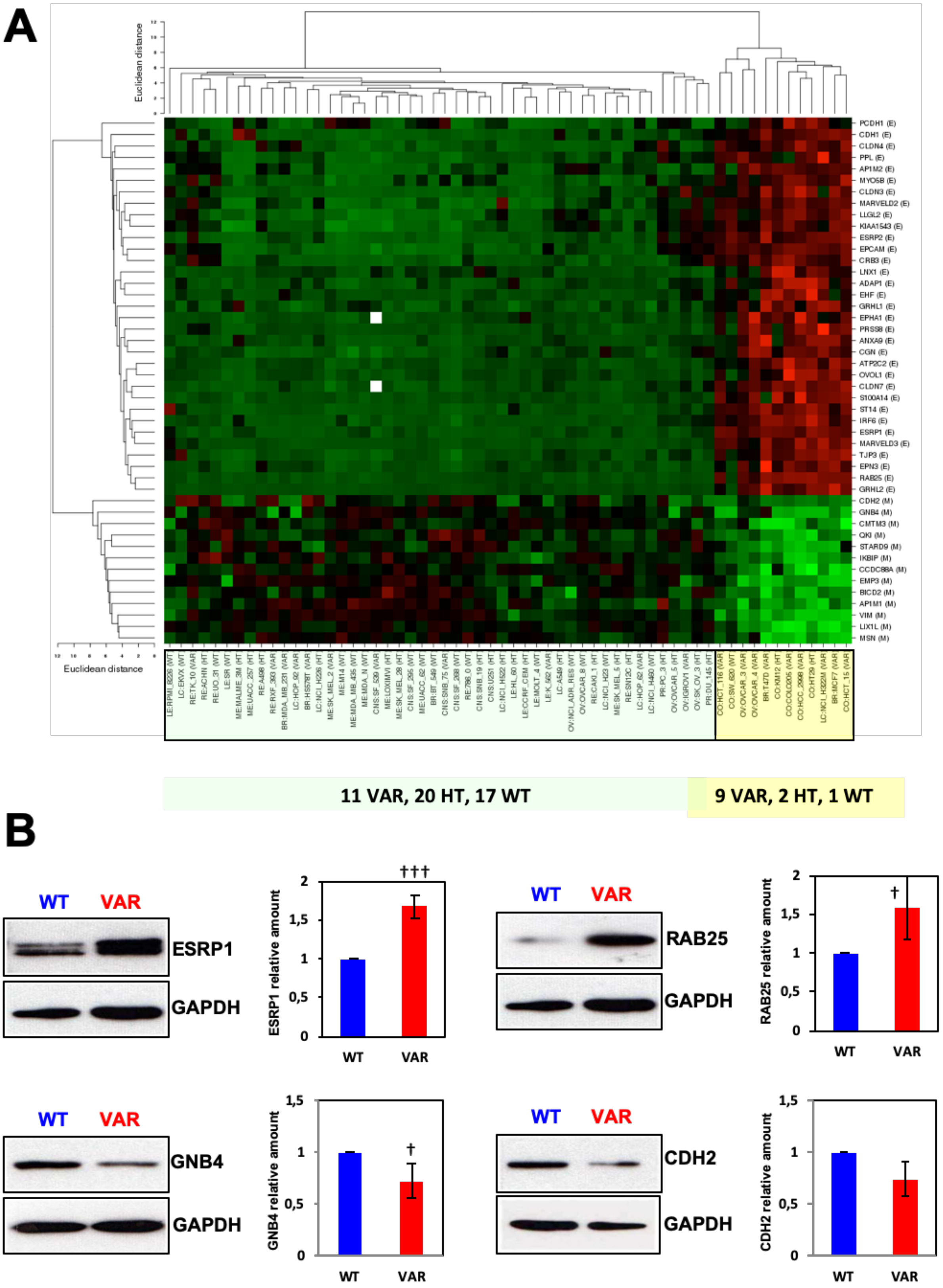

Supplemental Figure 3. The 3D structure of the protein CYP1B1 as extracted from the UniProt database [27]

The *N*- and *C*-terminal aminoacids are indicated, as well as the location of aminoacid #432 and the catalytic center (arrow).

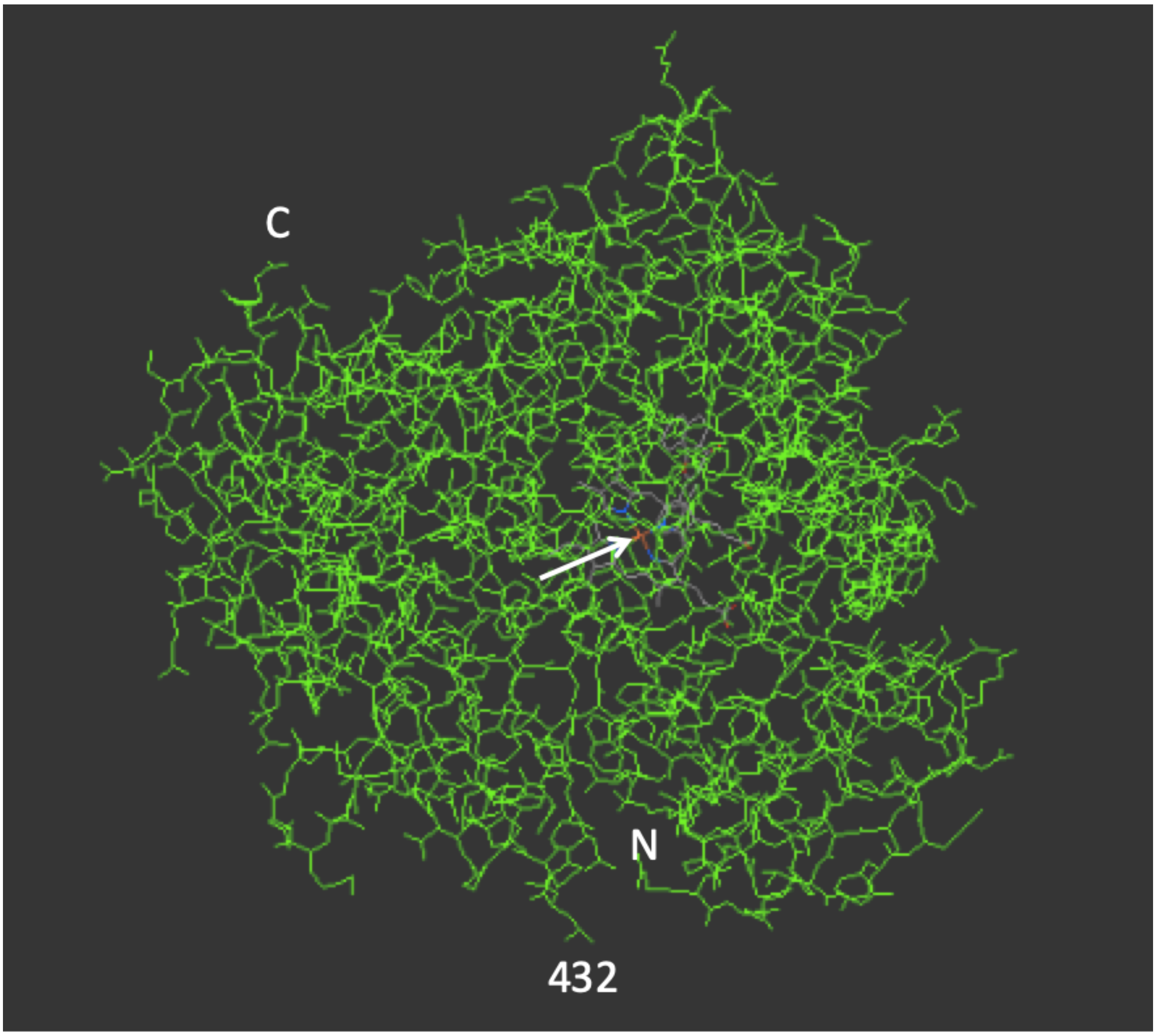

Supplement: Supplementary file 1 — Supplementary material [file 41416_2020_932_MOESM1_ESM.pdf]
